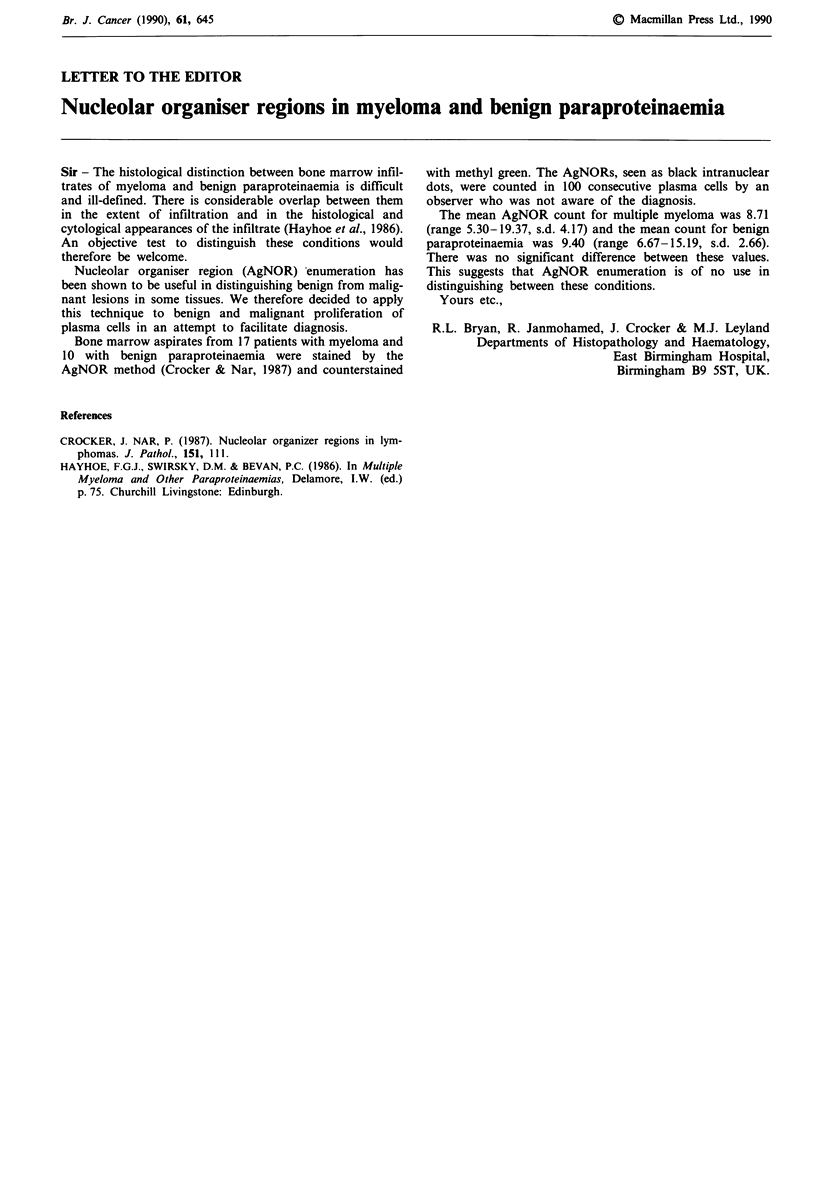# Nucleolar organiser regions in myeloma and benign paraproteinaemia.

**DOI:** 10.1038/bjc.1990.144

**Published:** 1990-04

**Authors:** R. L. Bryan, R. Janmohamed, J. Crocker, M. J. Leyland


*Br. J. Cancer* (1990), **61**, 645 © Macmillan Press Ltd., 1990

LETTER TO THE EDITOR

### Nucleolar organiser regions in myeloma and benign paraproteinaemia

**Sir** – The histological distinction between bone marrow infiltrates of myeloma and benign paraproteinaemia is difficult and ill-defined. There is considerable overlap between them in the extent of infiltration and in the histological and cytological appearances of the infiltrate (Hayhoe *et al.*, 1986). An objective test to distinguish these conditions would therefore be welcome.

Nucleolar organiser region (AgNOR) enumeration has been shown to be useful in distinguishing benign from malignant lesions in some tissues. We therefore decided to apply this technique to benign and malignant proliferation of plasma cells in an attempt to facilitate diagnosis.

Bone marrow aspirates from 17 patients with myeloma and 10 with benign paraproteinaemia were stained by the AgNOR method (Crocker & Nar, 1987) and counterstained with methyl green. The AgNORs, seen as black intranuclear dots, were counted in 100 consecutive plasma cells by an observer who was not aware of the diagnosis.

The mean AgNOR count for multiple myeloma was 8.71 (range 5.30–19.37, s.d. 4.17) and the mean count for benign paraproteinaemia was 9.40 (range 6.67–15.19, s.d. 2.66). There was no significant difference between these values. This suggests that AgNOR enumeration is of no use in distinguishing between these conditions.

Yours etc.,

R.L. Bryan, R. Janmohamed, J. Crocker & M.J. Leyland
Departments of Histopathology and Haematology,
East Birmingham Hospital,
Birmingham B9 5ST, UK.

**References**

CROCKER, J. NAR, P. (1987). Nucleolar organizer regions in lymphomas. *J. Pathol.*, **151**, 111.

HAYHOE, F.G.J., SWIRSKY, D.M. & BEVAN, P.C. (1986). In *Multiple Myeloma and Other Paraproteinaemias*, Delamore, I.W. (ed.) p. 75. Churchill Livingstone: Edinburgh.